# Supplementary material for: Using an artificial neural network to develop an optimal model of straight punch in boxing and training in punch techniques based on this model and real-time feedback
Source: PLoS One. 2021 Nov 29;16(11):e0259457. doi: 10.1371/journal.pone.0259457 (PMC8629283; doi:10.1371/journal.pone.0259457)
Supplement: Attachment — Submitted filename: Response to the review.docx [file pone.0259457.s001.docx]

Journal Requirements:

1. **Please ensure that your manuscript meets PLOS ONE's style requirements, including those for file naming.**

The article was formatted in accordance with the PLOS LaTex Template https://journals.plos.org/plosone/s/latex. I will attach a LaTex file as well.

2. **Please provide additional details regarding participant consent. In the ethics statement in the Methods and online submission information, please ensure that you have specified (1) whether consent was informed and (2) what type you obtained (for instance, written or verbal, and if verbal, how it was documented and witnessed). If your study included minors, state whether you obtained consent from parents or guardians. If the need for consent was waived by the ethics committee, please include this information.**

**If you are reporting a retrospective study of medical records or archived samples, please ensure that you have discussed whether all data were fully anonymized before you accessed them and/or whether the IRB or ethics committee waived the requirement for informed consent. If patients provided informed written consent to have data from their medical records used in research, please include this information.**

The research was approved by the University's ethics committee. All participants of the study reached the age of majority and gave written consent to participate in the study. This information will be included in the Cover Letter.

3. **We note that you have stated that you will provide repository information for your data at acceptance. Should your manuscript be accepted for publication, we will hold it until you provide the relevant accession numbers or DOIs necessary to access your data. If you wish to make changes to your Data Availability statement, please describe these changes in your cover letter and we will update your Data Availability statement to reflect the information you provide.**

I have granted access to github, where the research datasets are stored.

4. **Please clarify the sources of funding (financial or material support) for your study. List the grants or organizations that supported your study, including funding received from your institution.**

“The authors received no specific funding for this work.”

5. **Please complete your Competing Interests on the online submission form to state any Competing Interests.**

Yes, it will be done

**Review Comments to the Author**

In general, we would like to thank the reviewers, whose comments helped us to take a fresh look at our work and significantly improve it.

**Reviewer #1:**

**1. Introduction section can be extended to add the issues in the context of the existing work.**

**2. Literature review techniques have to be strengthened by including the issues in the current system and how the author proposes to overcome the same.**

**4. Research gaps, objectives of the proposed work should be clearly justified.**

These are interrelated questions and comments. We agree with all the comments, so we have completely redone the structure and content of the introduction. Each hypothesis, goal, and choice of research methods is justified by the work of other authors in this field.

**3. What is the motivation of the proposed work?**

Sports science is actively developing, while training in the technique of punches in boxing is still intuitive. The student imitates the movements of the teacher and this process does not yet have measurable parameters. Research on the biomechanics of punches almost does not help coaches, you need a model that fits well into coaching practice.

5. **The authors should consider more recent research done in the field of their study (especially in the years 2018 and 2020 onwards).**

We have extended the list of peer-reviewed papers in this area, especially with articles published over the past year.

**6. The paper needs to provide significant experimental details to correctly assess its contribution: What is the validation procedure used?**

We have supplemented the article K-fold method of cross-validation of the model. We also supplemented the article with statistical data verification.

**7. Kindly provide several references to substantiate the claim made in the abstract (that is, provide references to other groups who do or have done research in this area).**

The abstract has been significantly revised and expanded, and it is limited to 300 words, so references to similar works are included in the sections "Introduction" and " Results and discussion".

**8. An error and statistical analysis of data should be performed.**

We supplemented the article with statistical data verification. Statistical analysis was performed in Statistica, a software package for statistical analysis developed by StatSoft, which implements the functions of data analysis, data management, data mining, data visualization using statistical methods.

The average value of the series punches and the confidence interval 0.95 were calculated.

**9. The conclusion should state scope for future work.**

**10. Discuss the future plans with respect to the research state of progress and its limitations.**

Very good point. In the studies, raw IMUs data was used as a dataset, and in further studies, it is planned to apply pre-processing of the data (normalization, reduction of the dimension of the input data, etc.).

Only a straight boxing punch was chosen for the study, as it is the simplest in terms of coordination characteristics. However, based on the study, it can be assumed that for other boxing punches (hook, swing, uppercut), it will also be possible to develop an optimal model. Therefore, it is planned to conduct research in order to develop an optimal model of other boxing punches.

**11. Kindly refer the below paper:**

**1. Rajput, D.S., Basha, S.M., Xin, Q. et al. Providing diagnosis on diabetes using cloud computing environment to the people living in rural areas of India. J Ambient Intell Human Comput (2021).** [**https://doi.org/10.1007/s12652-021-03154-4**](https://doi.org/10.1007/s12652-021-03154-4)

A useful article, we have included it in the work.

**Reviewer #2:**

**Introduction lacks contribution and structure is missing**

We have completely redone the structure and content of the introduction. Each hypothesis, goal, and choice of research methods is justified by the work of other authors in this field.

**Figures need to be improved**

All figures have a resolution of 600 dpi, in accordance with the requirements of PLOS ONE.

**You need to top up the literature review**

We have extended the list of peer-reviewed papers in this area, especially with articles published over the past year.

**suggested should be applied https://ieeexplore.ieee.org/abstract/document/9430519 and Data collection method** [**https://www.sciencedirect.com/science/article/abs/pii/S0140366420318442**](https://www.sciencedirect.com/science/article/abs/pii/S0140366420318442)

A useful article, we have included it in the work.

**Conclusion should be improved**

We have reworked the conclusion, including our future research plans in this area.

**grammar should be improved**

We tried to improve the grammar of the article
